# Cyclodextrin-grafted polymers functionalized with phosphanes: a new tool for aqueous organometallic catalysis

**DOI:** 10.3762/bjoc.10.276

**Published:** 2014-11-11

**Authors:** Jonathan Potier, Stéphane Menuel, David Mathiron, Véronique Bonnet, Frédéric Hapiot, Eric Monflier

**Affiliations:** 1 Université Lille Nord de France, CNRS UMR 8181, Unité de Catalyse et de Chimie du Solide, UCCS Artois, Faculté Jean Perrin, rue Jean Souvraz, SP18, 62307 Lens Cédex, France; 2 Laboratoire des Glucides FRE CNRS 3517, 33 rue St Leu, 80039 Amiens, France

**Keywords:** biphasic catalysis, cyclodextrin, hydroformylation, phosphane, polymer

## Abstract

New cyclodextrin (CD)-grafted polymers functionalized with water-soluble phosphanes were synthesized in three steps starting from polyNAS. Once characterized by NMR spectroscopy and size-exclusion chromatography, they were used as additives in Rh-catalyzed hydroformylation of 1-hexadecene. The combined supramolecular and coordinating properties of these polymers allowed increasing the catalytic activity of the reaction without affecting the selectivities.

## Introduction

Although aqueous organometallic catalysis has been developed long before P. T. Anastas and J. C. Warner set out the foundations of Green Chemistry [1], the very essence of this concept relies on several of the twelve fundamental principles. As such, the use of effective catalysts and water as a solvent greatly contribute to the development of eco-friendly industrial processes. The most impressive example of industrial application that makes use of aqueous organometallic catalysis is undoubtedly the Ruhrchemie/Rhône-Poulenc process which allows conversion of propene mainly into linear butyraldehyde using a rhodium catalyst immobilized in the aqueous phase by coordination of the famous water-soluble ligand TPPTS (trisodium salt of the trisulfonated triphenylphosphane) [2]. However, while propene is partially soluble in water, terminal alkenes containing more than 6 carbon atoms cannot be converted using this process due to obvious insolubility problems. To overcome mass transfer limitations occurring between the catalyst-containing aqueous phase and the substrate-containing organic phase, various solutions have been implemented. For example, other solvents such as supercritical CO_2_, ionic liquids or fluorous phases have been used to immobilize the organometallic catalyst [3–5]. Co-solvents, surfactants, amphiphilic phosphanes, molecular receptors, polymers or dispersed particles have also been investigated to favour contacts between the aqueous and the organic compartments [6]. We especially developed biphasic catalytic systems in which cyclodextrin (CD) derivatives acted as interfacial additives [7–8]. These torus-like macrorings proved to be appropriate to supramolecularly recognize C8–C10 alkenes within their cavity and convert them into their corresponding aldehydes. Recently, the question arouse about alkenes containing more than 10 carbons. Effective solutions were thus developed using CD-dimers [9], CD-based hydrogels [10–11] and CD-based polymers [12]. In the present study, the concept has been taken a step further. While the CD-based polymer and TPPTS were added separately in the aqueous catalytic solution in our previous study, we synthesized a CD-substituted polymer functionalized with water-soluble phosphanes. The idea was to increase the local concentration of interfacial additive and phosphane-coordinated Rh catalyst at the aqueous/oganic interface to favour the substrate conversion. Herein, we detailed the synthesis and characterisation of this polymer and its catalytic behaviour in Rh-catalyzed hydroformylation of 1-hexadecene.

## Results and Discussion

**Scheme 1:**
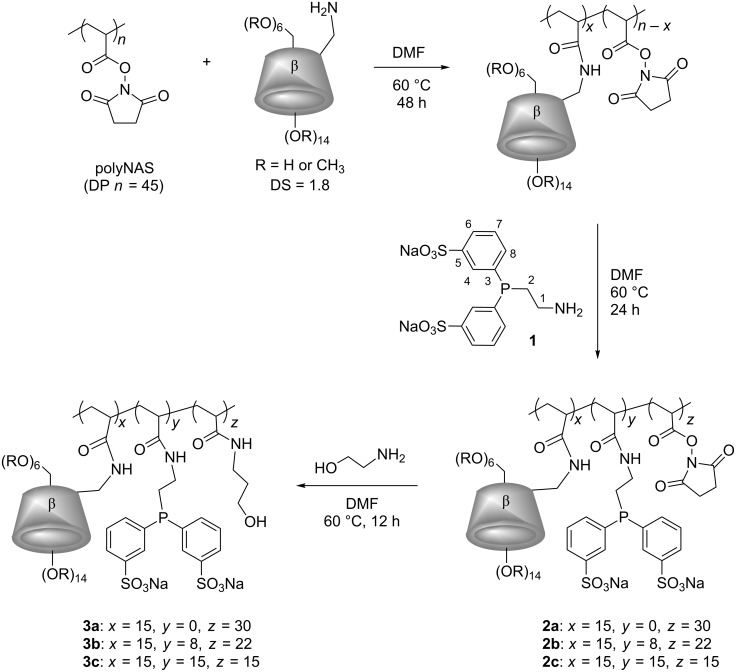
Synthesis of CD-substituted polymers **3a**, **3b** and **3c**.

A wide range of CD-based polymers have already been described in the literature [13–18]. To access the expected CD-substituted polymer functionalized with water-soluble phosphanes, a sulfonation step of a commercially available phosphane was first required. 2-(Diphenylphosphino)ethanamine was sulfonated in an oleum/H_2_SO_4_ mixture at room temperature over a period of 15 days. Once cold distilled water and trioctylamine (dissolved in chloroform) have been added, phosphane **1** was fractionally collected using a diluted solution of NaOH. The fractionalization allowed for a step-by-step removal of the phosphane oxide. After work-up, the sulfonated phosphane **1** (Scheme 1) was isolated in 55% yield as white crystals. The *meta*-sulfonation on the aromatic rings was confirmed by NMR. The COSY spectrum (Supporting Information File 1) was especially indicative of the *meta*-substitution. Indeed, H4 (Scheme 1) did not correlate with any other proton and appeared as an upfield shifted signal due to the electro-withdrawing effect of both the sulfonate group and the phosphorus. Moreover, H4 was detected as a doublet due to the ^3^*J* scalar coupling with the phosphorus. H6 also appeared as a doublet (due to the coupling with H7) but its resonance was a little bit more shielded than H4 as H6 did not benefit from the cumulative electron-withdrawing effects of both the sulfonate and the phosphorus. Contrary to H4, H6 showed cross-peaks with H7 and H8 whose doublets of doublets overlapped in the ^1^H spectrum and strongly correlated in the COSY spectrum.

Once the sulfonated phosphine synthesized, a polyNAS sample (DP *n* = 45) [10] reacted with the mono-amino randomly methylated β-CD (RAME-β-CD-NH_2_) [10] in DMF at 60 °C for 48 h under vigorous stirring (1500 rounds per minutes). The CD-substituted polymer then reacted with **1** in DMF at 60 °C for 24 h. Two different CD/**1** ratios have been considered for comparison with systems where the water-soluble phosphane and the polymer were added separately. Depending on the amount of **1**, CD-substituted phosphane-functionalized polymers **2b** and **2c** (Scheme 1) were obtained. To ensure the water solubility of the resulting polymers and remove the remaining succinimide groups from the polymer chains, a subsequent reaction of **2b** and **2c** with aminoethanol for 12 h in the same experimental conditions led to the trisubstituted polymers **3b** and **3c** respectively (Scheme 1) in 85% yield as pale yellow powders.

**Figure 1:**
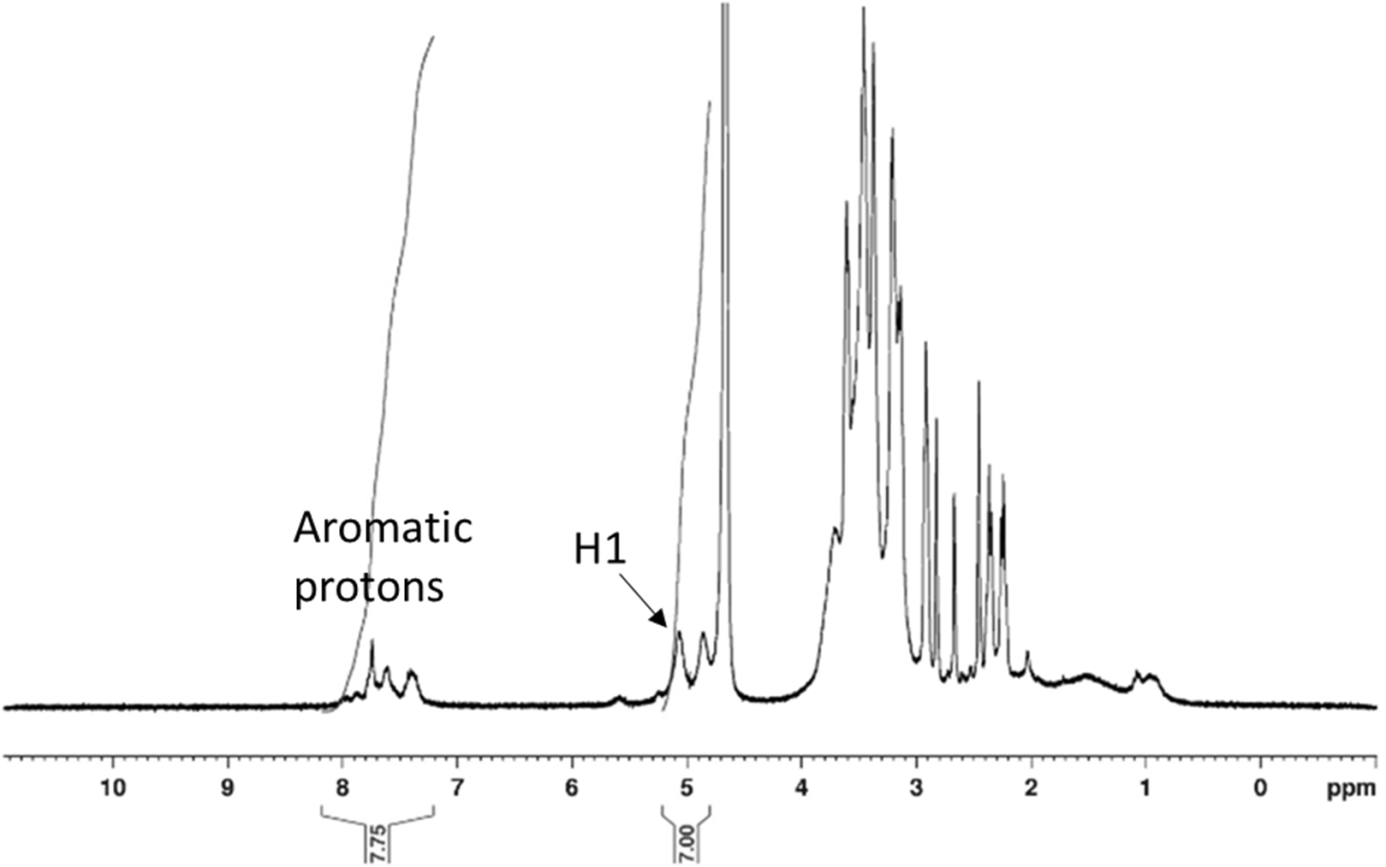
^1^H NMR spectrum of **3c** in D_2_O at 25 °C.

Polymers **3a**, **3b** and **3c** were characterized by ^1^H NMR both in D_2_O and DMF-*d*_7_. Integration of the polymer chain protons and the H-1 CD protons showed that 33% CDs were grafted onto the polymer chains of **3b** and **3c**. Integration of the H-1 CD protons and the aromatic protons of **1** in D_2_O allowed confirming the CD/phosphane ratio onto polymers **3b** and **3c** (Figure 1).

**Figure 2:**
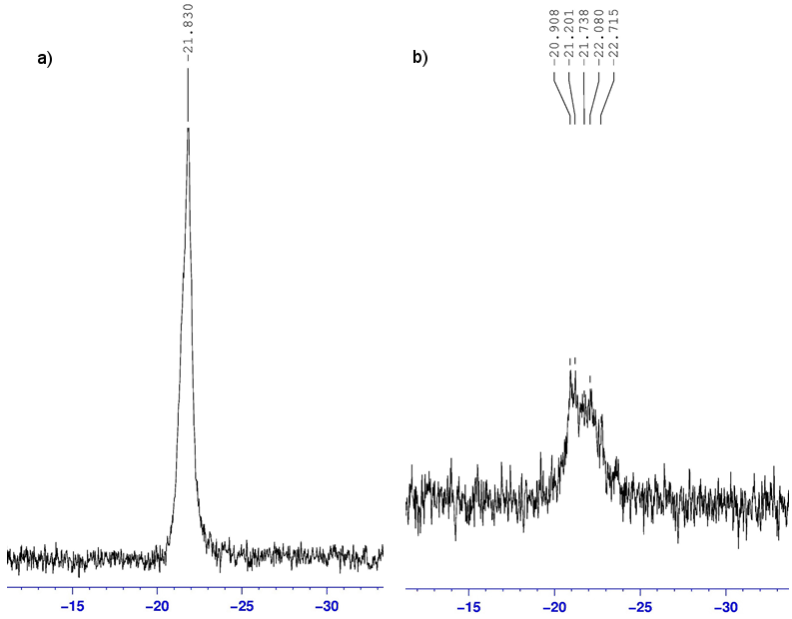
^31^P NMR spectrum of **3b** in a) DMF-*d*_7_ at 25 °C and b) D_2_O at 25 °C.

**Figure 3:**
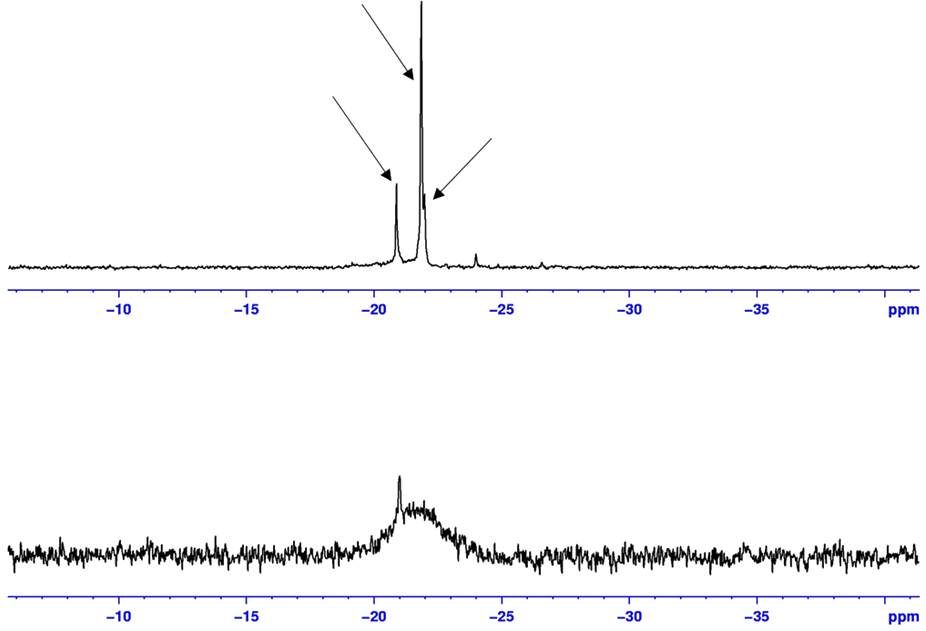
^1^H NMR spectrum of **3b** (10 mM) in D_2_O at 25 °C (below) and 60 °C (above).

16.5% and 33% phosphanes were also incorporated onto **3b** and **3c**, respectively. The purity of the phosphane-containing polymers **3b** and **3c** was subsequently confirmed by ^31^P NMR. Spectra of **3b** (Figure 2a) and **3c** recorded in DMF-*d*_7_ clearly revealed a broad ^31^P resonance (2 ppm wide) indicative of different chemical environments of the phosphorus. The broadening of the ^31^P NMR signal was even more marked in D_2_O (Figure 2b). Several factors might be involved and be responsible for such disparity in phosphorus resonances. First, phosphanes could be included into CD cavities or not (“free” phosphanes). Moreover, as these polymers were mixtures of compounds, polymers differing from their chain length could have different chemical shifts.

In addition, each polymer chain contained three asymmetric carbon atoms, and therefore many diastereomers were present, all of them resulting in a slightly different ^31^P chemical shift. However, a simplification of the ^31^P spectra occurred at 60 °C. Only three peaks could be observed at −20.88, −21.85 and −21.98 ppm (Figure 3). We believed that the increase in temperature disfavoured the CD/phosphane inclusion complexes, thus greatly reducing the number of possible chemical environments. The remaining peaks could be attributed to three different diastereoisomers.

Note that, whatever the solvent, no resonance corresponding to **1** could be observed indicative of the total grafting of **1** onto the polymer chains (see Figure S5, (Supporting Information File 1) for details on a physical mixture of **1** and **3b** in DMF-*d*_7_).

To get further information on the exact conformations of **3b** and **3c** in D_2_O, 2D T-ROESY measurements have been performed to reveal potential interactions between the phosphane aromatic protons and the inner CD protons H-3 and H-5. None of the spectra revealed any correlation between these protons. Indeed, as the peaks were wide (as usually observed for polymers), the NMR signals were drowned in the background noise (under the detection limit). However, an inclusion of the phosphane moiety within the CD cavity could not be excluded at this stage as a 2D T-ROESY spectrum of a 1/5 mixture of RAME-β-CD and **1** clearly indicated cross-peaks between **1** and the inner protons of RAME-β-CD (see Supporting Information File 1). Knowing that sulfonated phosphanes could by partially included into CD cavities, DOSY experiments were then carried out to get insights on the intramolecular interactions existing between the grafted phosphanes and the grafted CDs of **3b**. To evaluate the strength of the interaction, RAME-β-CD was added as a competitor in the solution (Supporting Information File 1). In DMSO-*d*_6_, RAME-β-CD and **3b** showed different diffusion coefficient (*D* = 1.78.10^−10^ and 8.05.10^−11^ m^2^/s, respectively) when they were analysed in two different NMR tubes. However, when they were mixed together, only one diffusion coefficient could be measured whatever the additional amount of RAME-β-CD, indicative of the interaction of RAME-β-CD with the polymer. Two different diffusion coefficients would have been observed otherwise. Similar behaviours were observed in D_2_O and CDCl_3_. Accordingly, the intramolecular interaction between the grafted phosphanes and the grafted CDs was not very strong as the recognition process between them could be easily displaced by a competitor.

**Figure 4:**
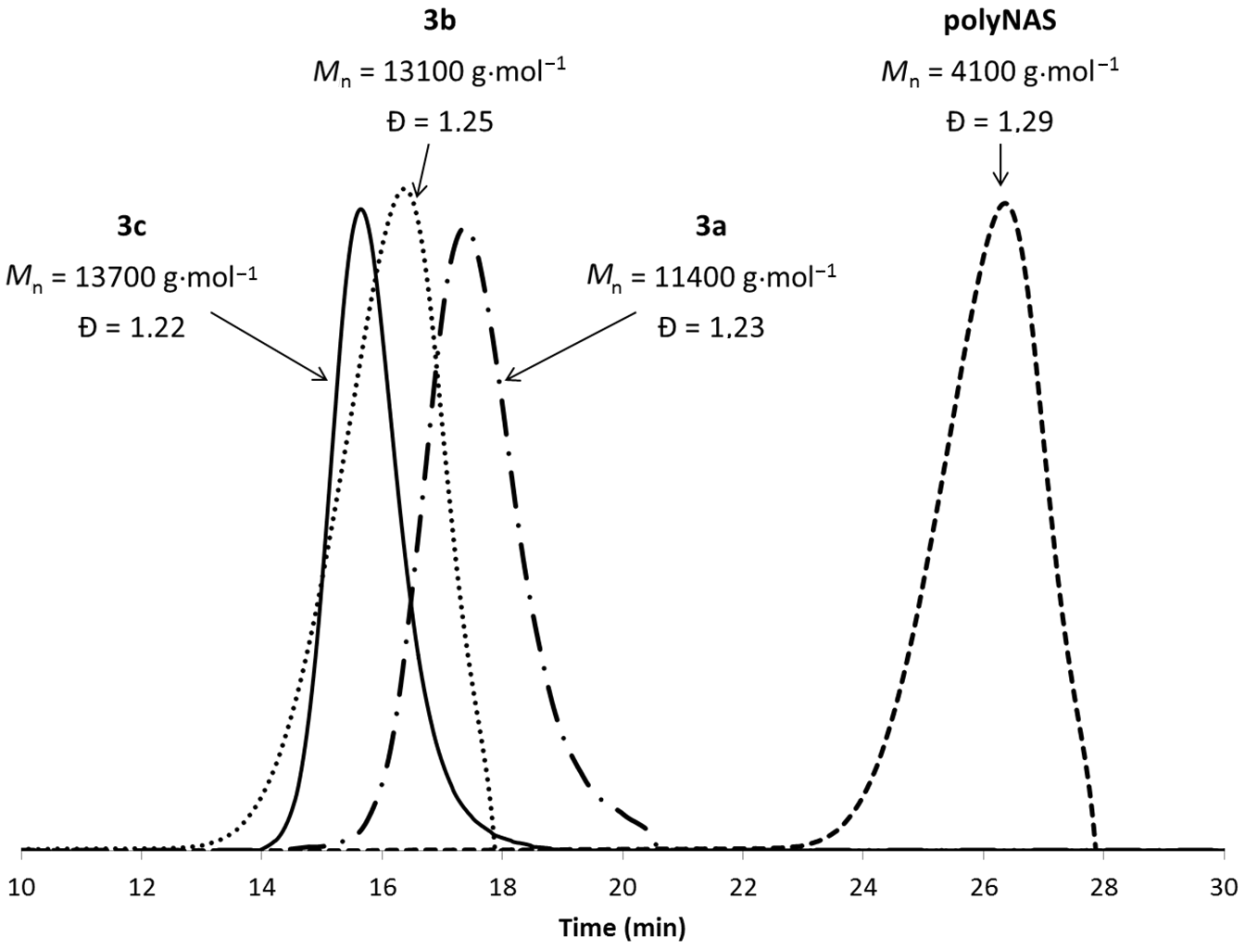
Size-exclusion chromatography of polyNAS and polymers **3a**, **3b** and **3c**.

The number average molecular weights *M*_n_ and dispersity Đ of **3b** and **3c** were determined by size-exclusion chromatography (SEC). *M*_n_ were 13100 and 13700 g·mol^−1^ and Đ were 1.25 and 1.22 for **3b** and **3c**, respectively (Figure 4). For comparison (see below the catalytic experiments); a CD-substituted polymer **3a** that did not contain any phosphane has also been synthesized (*M*_n_ = 11400 g·mol^−1^, Đ = 1.23).

The catalytic performances of polymers **3a**, **3b** and **3c** have been evaluated in a Rh-catalyzed hydroformylation of 1-hexadecene at 80 °C under 50 bar CO/H_2_ in a 1/1 stoichiometric ratio. The results were gathered in Table 1.

**Table 1:** Rh-catalyzed hydroformylation of 1-hexadecene.^a^

Entry	Polymer/phosphane	Conversion (%)	Chemoselectivity (% aldehydes)	Regioselectivity (l/b ratio)

1	**1**	3	28	2.7
2	**1** + RAME-β-CD	34	63	1.6
3	**1** + **3a**	81	61	1.8
4	**3b**	98	62	1.9
5	**3c**	8	57	1.7

^a^Conditions: Rh:substrate ratio: 1/140, Rh:phosphane ratio: 1/0 (**3a**), 1/5 (**3b**) or 1/10 (**3c**), 80 °C, 50 bar CO/H_2_, reaction time = 1 h. Conversions and selectivities were determined by GC and ^1^H NMR.

Compared to **1** all the synthesized CD-substituted polymers led to better activities and aldehyde selectivity. A slight decrease in the regioselectivity was also observed and corroborated previous studies dealing with the role of the CD at the aqueous/organic interface [10]. Effects of the phosphanes grafted onto the polymer backbone on the catalytic performances were much more intricate. Indeed, the conversion was greatly dependent upon the CD:phosphane ratio. A significant increase in the conversion (17%) was observed when comparing **3a** and **3b** (81 vs 98% conversion, respectively). These results highlighted the benefit resulting from the phosphane grafting onto the polymer backbone. Indeed, as the phosphane coordinated the metallic species, the catalyst and the CD were in close vicinity. Hence, when the substrate was supramolecularly recognized by the CD cavity, it could then rapidly react with the catalyst to be hydroformylated. The closeness of the protagonists (CD, catalyst and substrate) was clearly determining in this process. However, while a 2:1 CD/phosphane ratio had a positive effect on the catalytic activity, only 8% 1-hexadecene were converted using a stoichiometric CD/phosphane ratio. The explanation of such as difference between the 2:1 and 1:1 ratios lies in the supramolecular interaction existing between the CD cavity and **1**. Indeed, we previously demonstrated that, concurrently to the substrate inclusion, **1** could also be included within the CD cavities. While **3b** still had available CD cavities (twice more CDs than phosphanes) to recognize the substrate, the CD cavities of **3c** (equal number of CDs and phosphanes) were mainly occupied by the grafted phosphanes and could not efficiently recognize the substrate at the aqueous/organic interface. Excess CDs regarding **1** was thus required for the polymer to be effective in catalytic conditions.

## Conclusion

To sum up, we synthesized a new CD-grafted polymer functionalized with water-soluble phosphane moieties which acted as a very effective tool in the aqueous Rh-catalyzed hydroformylation of 1-hexadecene. Both the supramolecular properties of the CD and the coordination ability of the phosphane were combined into the same molecular object. During the course of the reaction, the closeness of the three main protagonists (substrate, CD, phosphane) led to a significant increase in the conversion compared to a catalytic system where the CD and the phosphane were not grafted on the same polymer chain. These interfacial polymer-based additives paved the way to the development of new catalytic systems for the conversion of very hydrophobic substrates.

## Experimental

### 2-(Bis(*m*-sulfonatophenyl)phosphino)ethanamine, sodium salt (**1**)


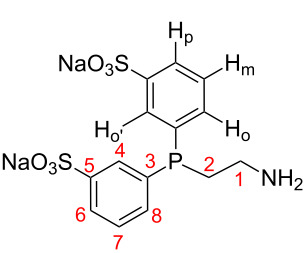


In a Schlenk tube were stirred 3.4 mL H_2_SO_4_ and 0.85 mL oleum at room temperature for 5 min. Once the solution has been degassed under nitrogen, degassed 2-(diphenylphosphino)ethanamine (2.0 g, 8.8 mmol) were then canulated on the acidic solution previously cooled in an ice bath. 7.1 mL oleum (4.4 equiv per phenyl moiety) were then added under nitrogen. The resulting solution was stirred at 800 rpm. Brought back to room temperature, the solution was stirred for 15 days. The Schlenk tube was then placed in an ice bath and 43 mL distilled water were added dropwise. The resulting solution was diluted in 200 mL cold water. 9.2 g (26 mmol) trioctylamine were added along with 45 mL chloroform. After the recovered organic phase has been washed with water, phosphane **1** was fractionally collected using a diluted solution of NaOH (400 µL NaOH 2 N in 4 mL water). Water was evaporated in a rotary evaporizer. 2.1 g of **1** were isolated as white crystals in 55% yield. ^31^P NMR (121.49 MHz, 21 °C, D_2_O) δ 36.27 (P=O, 5%), −21.48 (P, 95%); ^1^H NMR (300.13 MHz, 21 °C, D_2_O) δ 7.88 (d, *J* = 7.2 Hz, 2H, Hp), 7.78 (d, *J* = 7.8 Hz, 2H, H_o’_), 7.47–7.59 (m, 4H, H_m_ and H_o_), 3.03 (m, 2H, CH_2_(1)), 2.52 (m, 2H, CH_2_(2)); ^13^C{^1^H} NMR (75.5 MHz, 21 °C, D_2_O) δ 142.7 (d, ^3^*J*(P,C) = 6.20 Hz, C5), 136.8 (d, ^1^*J*(P,C) = 11.93 Hz, C7), 135.7 (d, ^2^*J*(P,C) = 19.49 Hz, C8), 129.54 (d, ^3^*J*(P,C) = 6.50 Hz, C3), 128.84 (d, ^2^*J*(P,C) = 20.21 Hz, C4), 126.48 (s, C6), 36.54 (d, ^2^*J*(P,C) = 25.86 Hz, C2), 24.74 (d, ^1^*J*(P,C) = 12.93 Hz, C1).

### PolyNAS functionalization

In a Schlenk tube were degassed 1.5 g (1.1 mmol) RAME-β-CD-NH_2_ and 558 mg (3.3 mmol) polyNAS. 20 mL degassed anhydrous DMF were canulated into the tube. The solution was vigorously stirred at 1500 rpm under nitrogen at 60 °C for 48 h. the solution was then canulated into another Schlenk tube containing 238 mg (0.55 mmol) or 476 mg (1.1 mmol) phosphane **1** to form polymers **2b** and **2c**, respectively. The resulting solution was stirred at 1500 rpm under nitrogen at 60 °C for another 24 h. Eventually, 403 mg (6.6 mmol) degassed aminoethanol were added dropwise and the solution was stirred under nitrogen at 60 °C for 12 h. Polymers **3a**, **3b** and **3c** were precipitated in a degassed acetone/Et_2_O (1:1) mixture. Pale yellowish powders were isolated in 82–85% yield.

Polymer **3a**: ^1^H NMR (300.13 MHz, 21 °C, D_2_O) δ 5.06 (br. s, 3.5H), 4.87 (br. s, 3.5H), 3.75–3.60 (m, 12.3H), 3.60– 3.53 (m, 32.4H), 3.41 (br. s, 10.7H), 3.39 (br. s, 5.1H), 3.30–3.10 (m, 22.1 H), 2.31 (m, 4H, CH_2_), 2.22 (m, 4H, CH_2_), 1.53 (br. s, 3H), 0.95 (br. s, 6H); J-MOD NMR (75.5 MHz, 21 °C, D_2_O) δ 171.5, 168.8, 101.1, 98.7, 82.9–80.0, 77.4, 60.4, 58.8, 46.0, 41.8, 31.0, 25.5, 11.6.

Polymer **3b**: ^31^P NMR (121.49 MHz, 21 °C, D_2_O) δ 37.40–37.00 (br m, P=O, 6%), −21.84 (br m, P, 94%); ^1^H NMR (300.13 MHz, 21 °C, D_2_O) δ 7.91–7.25 (br. m, 4H), 5.08 (br. s, 3.5H), 4.89 (br. s, 3.5H), 3.75–3.56 (m, 12.3H), 3.58–3.52 (m, 32.4H), 3.44 (br. s, 10.7H), 3.42 (br. s, 5.1H), 3.30–3.10 (m, 22.1 H), 2.94 (m, 1H, CH_2_), 2.49 (m, 1H, CH_2_), 2.33 (m, 3H, CH_2_), 2.24 (m, 3H, CH_2_), 1.57 (br. s, 3H), 0.97 (br. s, 6H); J-MOD NMR (75.5 MHz, 21 °C, D_2_O) δ 171.7, 168.7, 165.5, 162.6, 101.4, 98.7, 82.8–80.0, 77.7, 60.5, 58.9, 46.0, 42.0, 31.2, 28.9, 25.8, 11.7.

Polymer **3c**: ^31^P NMR (121.49 MHz, 21 °C, D_2_O) δ 37.40–37.06 (br m, P=O, 6%), −21.81 (br m, P, 94%); ^1^H NMR (300.13 MHz, 21 °C, D_2_O) δ 7.96–7.30 (br. m, 8H), 5.07 (br. s, 3.5H), 4.86 (br. s, 3.5H), 3.74–3.61 (m, 12.3H), 3.59–3.55 (m, 32.4H), 3.46 (br. s, 10.7H), 3.38 (br. s, 5.1H), 3.31–3.13 (m, 22.1 H), 2.92 (m, 2H, CH_2_), 2.46 (m, 2H, CH_2_), 2.34 (m, 2H, CH_2_), 2.24 (m, 2H, CH_2_), 1.51 (br. s, 3H), 0.96 (br. s, 6H); J-MOD NMR (75.5 MHz, 21 °C, D_2_O) δ 171.6, 168.9, 165.3, 162.8, 101.4, 98.9, 82.6–80.1, 77.6, 60.3, 58.6, 46.1, 41.9, 31.0, 28.8, 25.7, 11.9.

### Catalytic experiments

Aqueous Rh-catalyzed hydroformylation: A mixture of Rh(CO)_2_(acac) (3 mg, 0.012 mmol. 1 equiv), alkene (1.63 mmol, 140 equiv), **1** (25 mg, 0.058 mmol, 5 equiv) and CDs (0.12 mmol, 10 equiv) or polymer **3a**, **3b** or **3c** (calculated for 10 equiv CDs) dissolved in water (6 mL) was degassed by three freeze-pump-thaw cycles and introduced in a previously purged autoclave. Once a temperature of 80 °C has been reached, the autoclave was pressurized under CO/H_2_ pressure (50 bar) and the solution was vigorously stirred (1500 rpm). When the reaction was over, the apparatus was allowed to cool to room temperature and depressurized. The organic phase was extracted using diethyl ether. After evaporation of diethyl ether under vacuum, the product was analyzed by GC and by ^1^H and ^13^C NMR experiments. All runs have been performed at least twice in order to ensure reproducibility.

## Supporting Information

File 1 NMR spectra.
