# Supplementary material for: Cyclodextrin-grafted polymers functionalized with phosphanes: a new tool for aqueous organometallic catalysis
Source: Beilstein J Org Chem. 2014 Nov 11;10:2642–8. doi: 10.3762/bjoc.10.276 (PMC4273264; doi:10.3762/bjoc.10.276)

Supporting Information

for

Cyclodextrin-grafted polymers functionalized with phosphanes: a new tool for aqueous organometallic catalysis

Jonathan Potier[1], Stéphane Menuel[1], David Mathiron[2], Véronique Bonnet[2], Frédéric Hapiot[1] and Eric Monflier*[1]

Address: [1]Université Lille Nord de France, CNRS UMR 8181, Unité de Catalyse et de Chimie du Solide, UCCS Artois, Faculté Jean Perrin, rue Jean Souvraz, SP18, 62307 Lens Cédex, France, [2]Laboratoire des Glucides FRE CNRS 3517, 33 rue St Leu 80039 Amiens, France.

Email: Eric Monflier - eric.monflier@univ-artois.fr

*Corresponding author

NMR spectra

	Page
**Figure S1.** 2D COSY spectrum of **1** (10 mM) in D_2O at 25 °C.	s3
**Figure S2.** 2D T-ROESY spectrum of a 1/5 mixture of RAME-β-CD (2 mM) and **1** (10 mM) in D_2O at 25 °C.	s4
**Figure S3.** ^{1}H NMR spectrum of **3b** (10 mM) in DMF-d_7 at 25 °C.	s5
**Figure S4.** ^{1}H NMR spectrum of **3c** (10 mM) in DMF-d_7 at 25 °C.	s6
**Figure S5.** ^{31}P NMR spectrum of a mixture of **1** (2 mM) and **3b** (10 mM) in DMF-d_7 at 25 °C.	s7
**Figure S6.** DOSY experiments of a **3b** (15 mg) and RAME-β-CD (15 mg) in DMSO-d6 (500 μL) at 25 °C.	s8
**Figure S7.** DOSY spectra of mixtures of **3b** (15 mg) and various amounts of RAME-β-CD in DMSO-d6 (500 μL) at 25 °C.	s9
**Figure S8.** DOSY spectra of **3b** (15 mg) and RAME-β-CD (15 mg) in D_2O (500 μL) at 25 °C.	s10
**Figure S9.** DOSY spectra of mixtures of **3b** (15 mg) and various amounts of RAME-β-CD in D_2O (500 μL) at 25 °C.	s11
**Figure S10.** DOSY spectra of **3b** (15 mg) and RAME-β-CD (15 mg) in $CDCl_3$ (500 μL) at 25 °C.	s12
**Figure S11.** DOSY spectra of **3b** (15 mg) and a mixture of **3b** (15 mg) and RAME-β-CD (15 mg) in $CDCl_3$ (500 μL) at 25 °C.	s13

Figure S1. 2D COSY spectrum of **1** (10 mM) in D_2O at 25 °C.

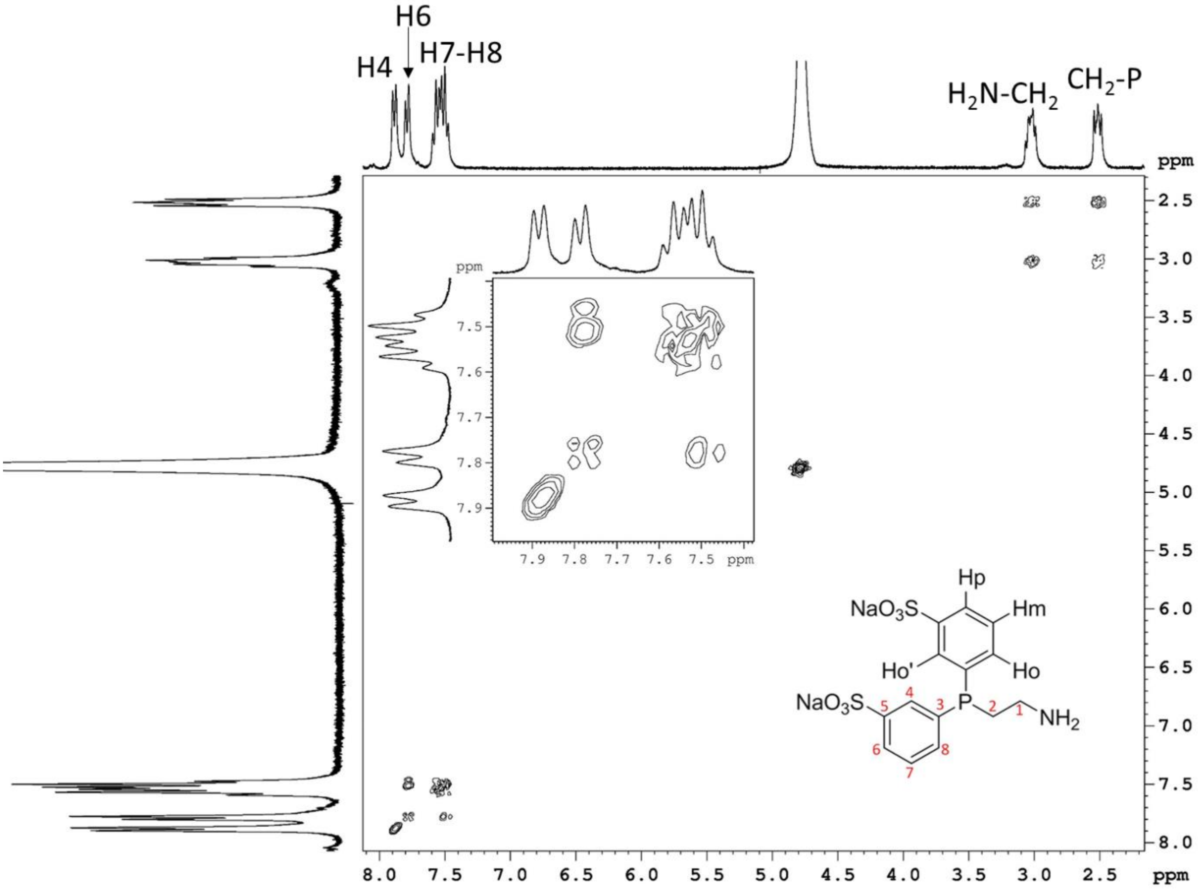

Figure S2. 2D T-ROESY spectrum of a 1/5 mixture of RAME-β-CD (2 mM) and **1** (10 mM) in D_2O at 25 °C.

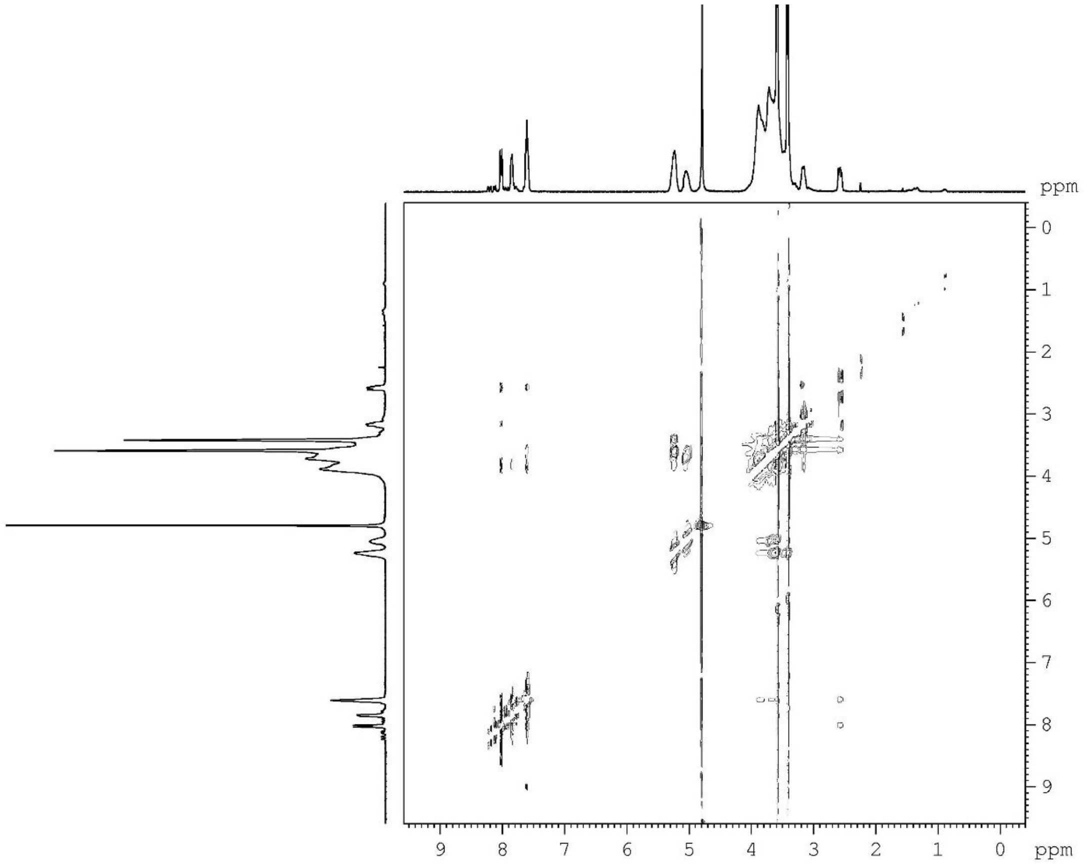

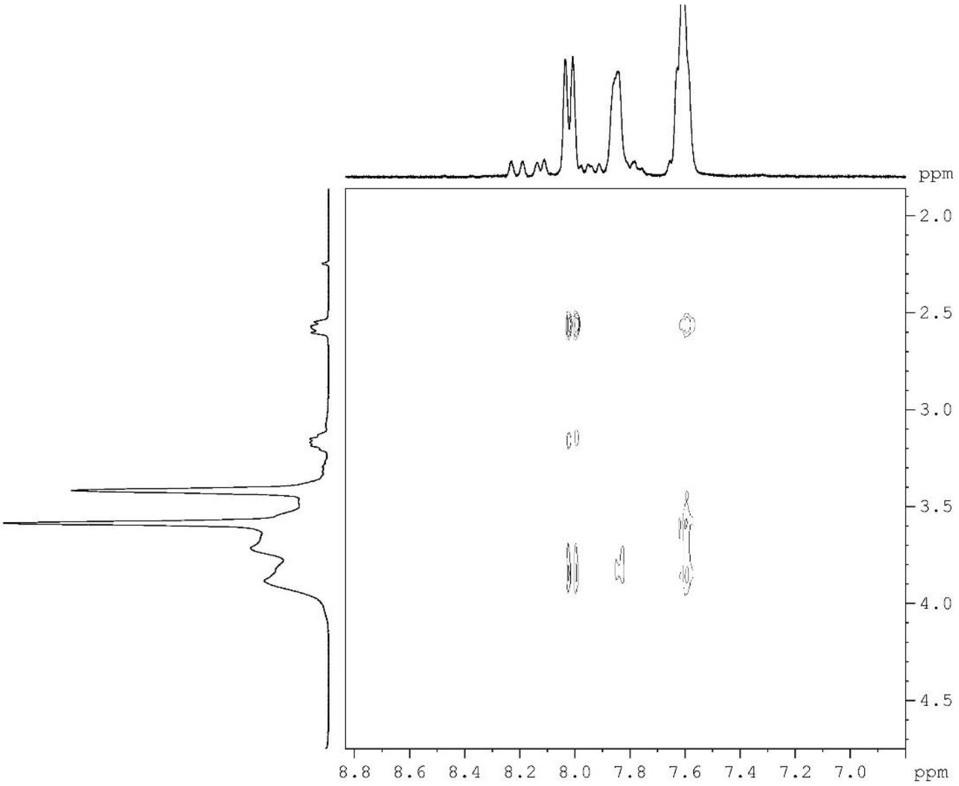

Figure S3. ^{1}H NMR spectrum of **3b** (10 mM) in DMF-d_7 at 25 °C.

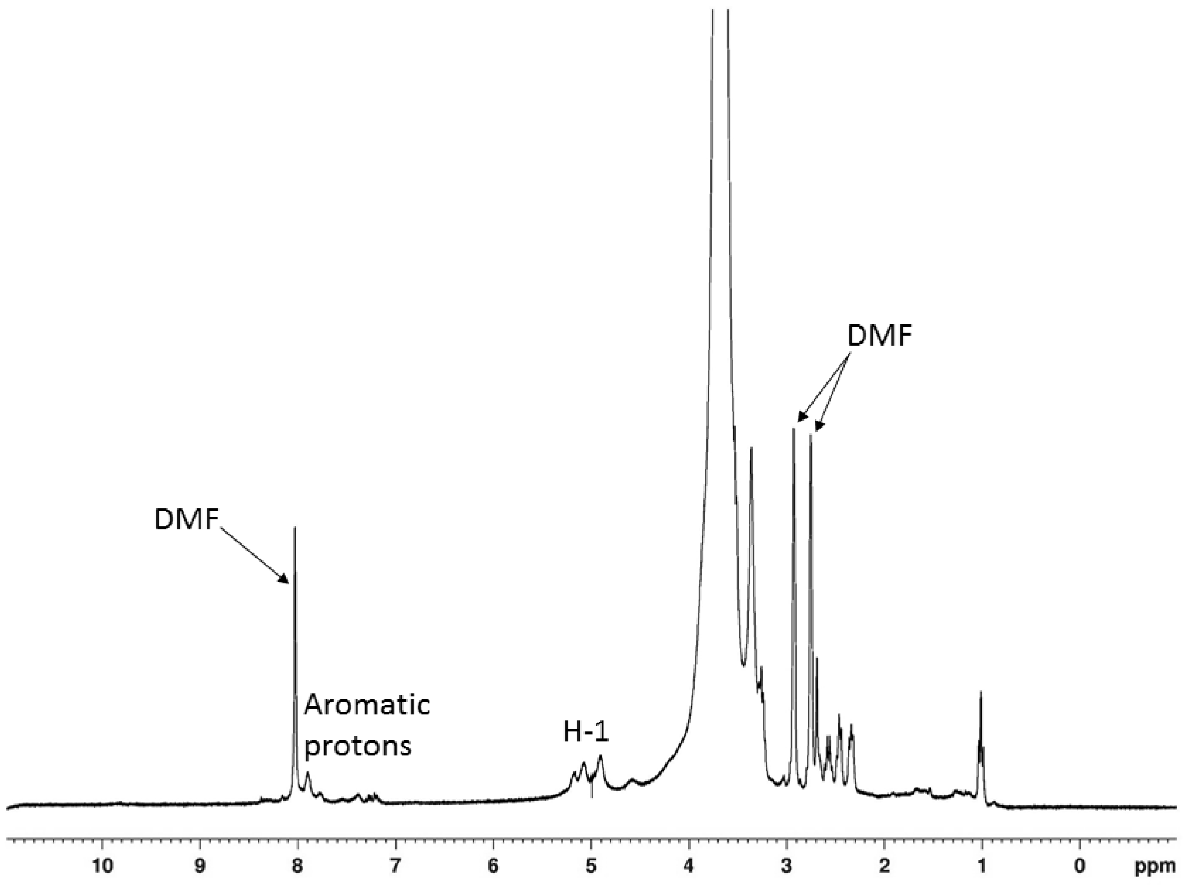

Figure S4. ^{1}H NMR spectrum of **3c** (10 mM) in DMF-d_7 at 25 °C.

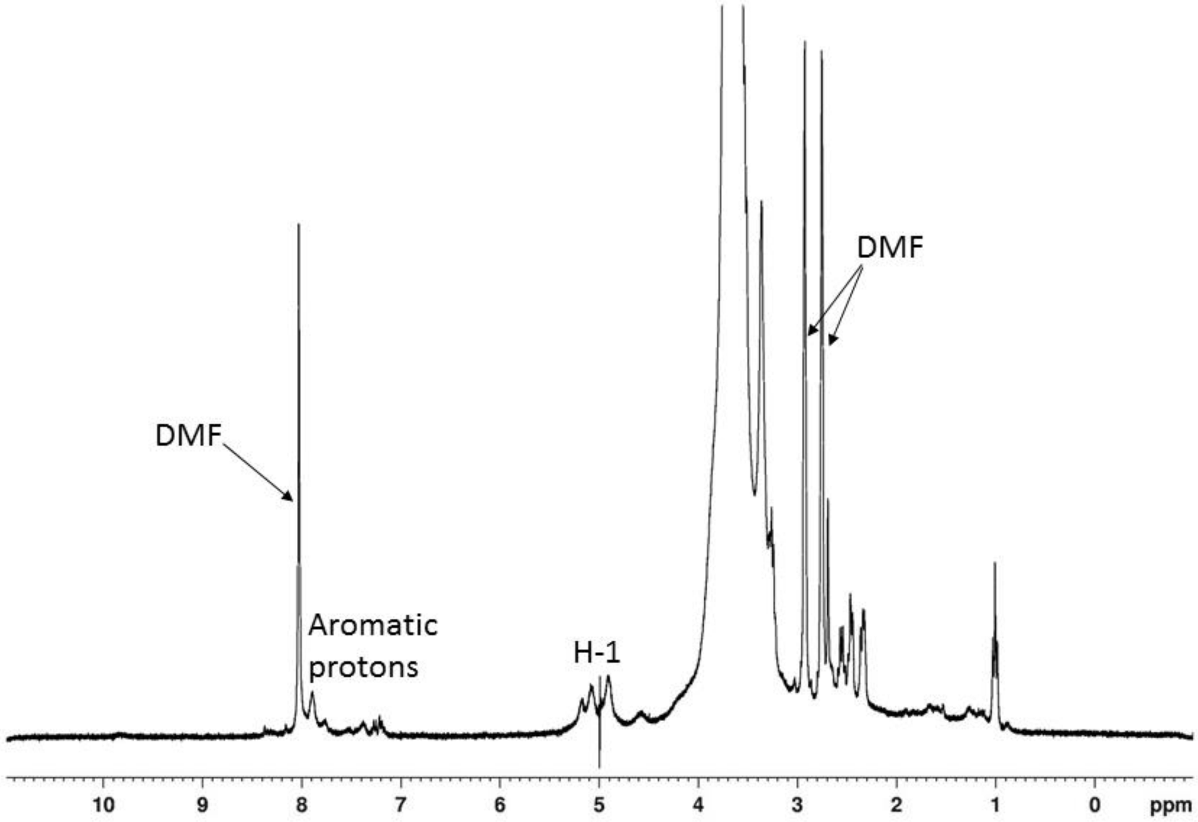

Figure S5. ^{31}P NMR spectrum of a mixture of **1** (2 mM) and **3b** (10 mM) in DMF-d_7 at 25 °C.

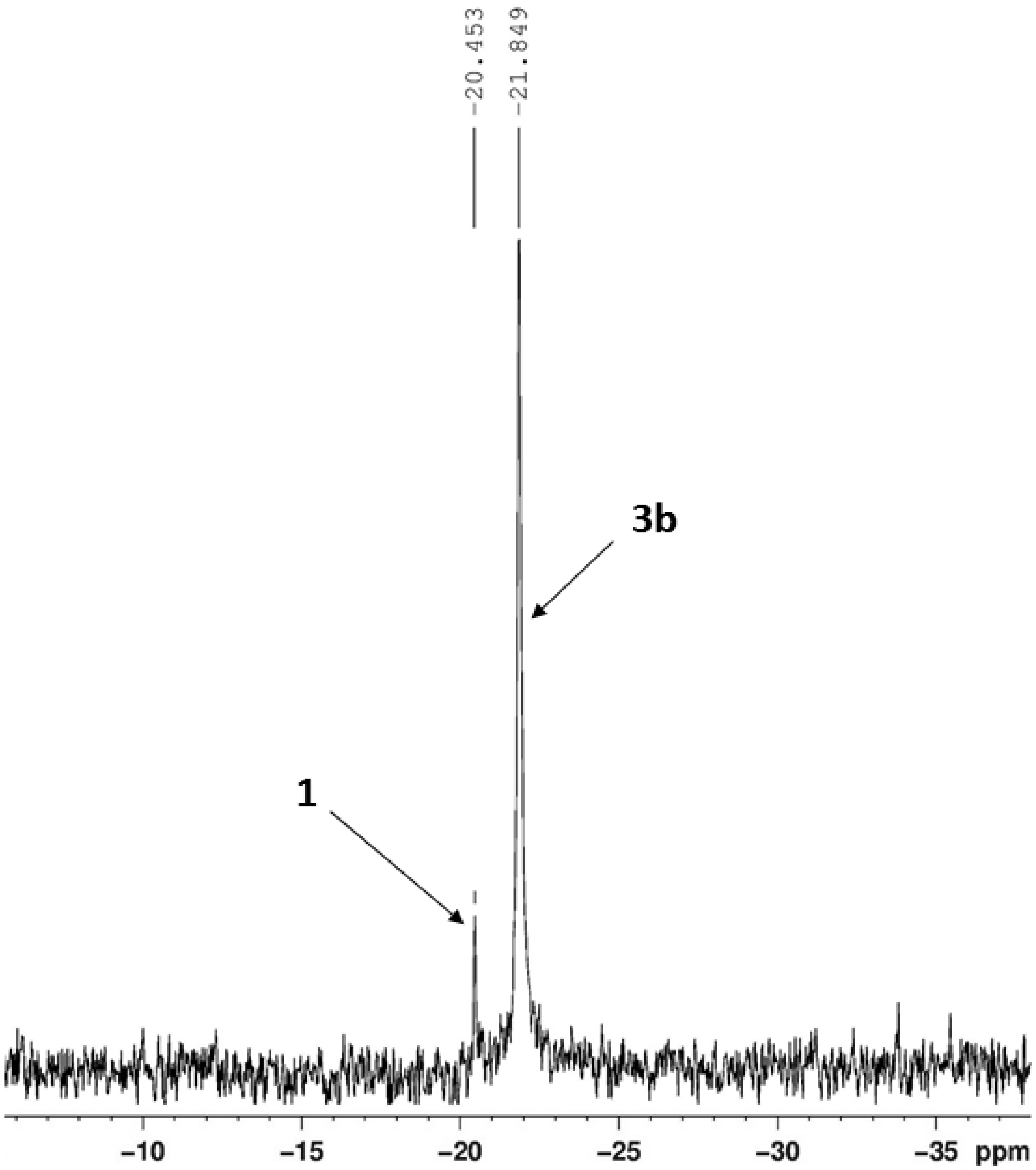

Figure S6. DOSY experiments of a **3b** (15 mg) and RAME-β-CD (15 mg) in DMSO-d6 (500 μL) at 25 °C.

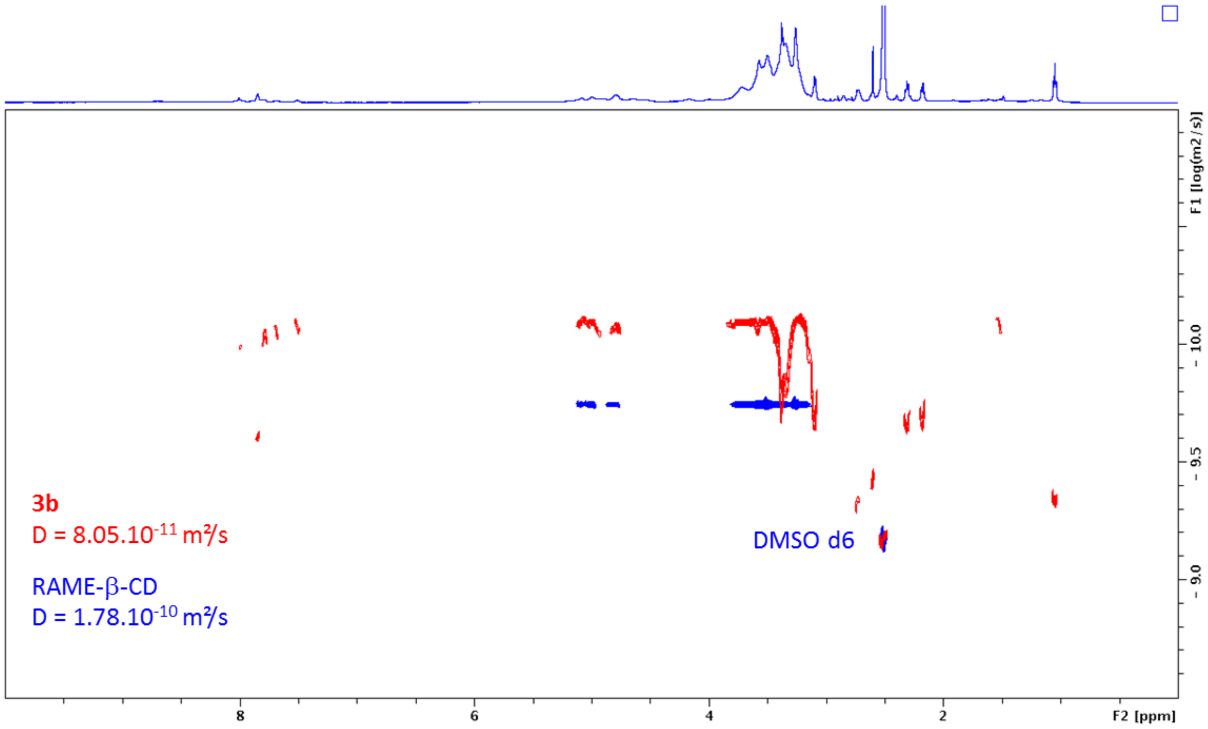

Figure S7. DOSY spectra of mixtures of **3b** (15 mg) and various amounts of RAME-β-CD in DMSO-d6 (500 μL) at 25 °C.

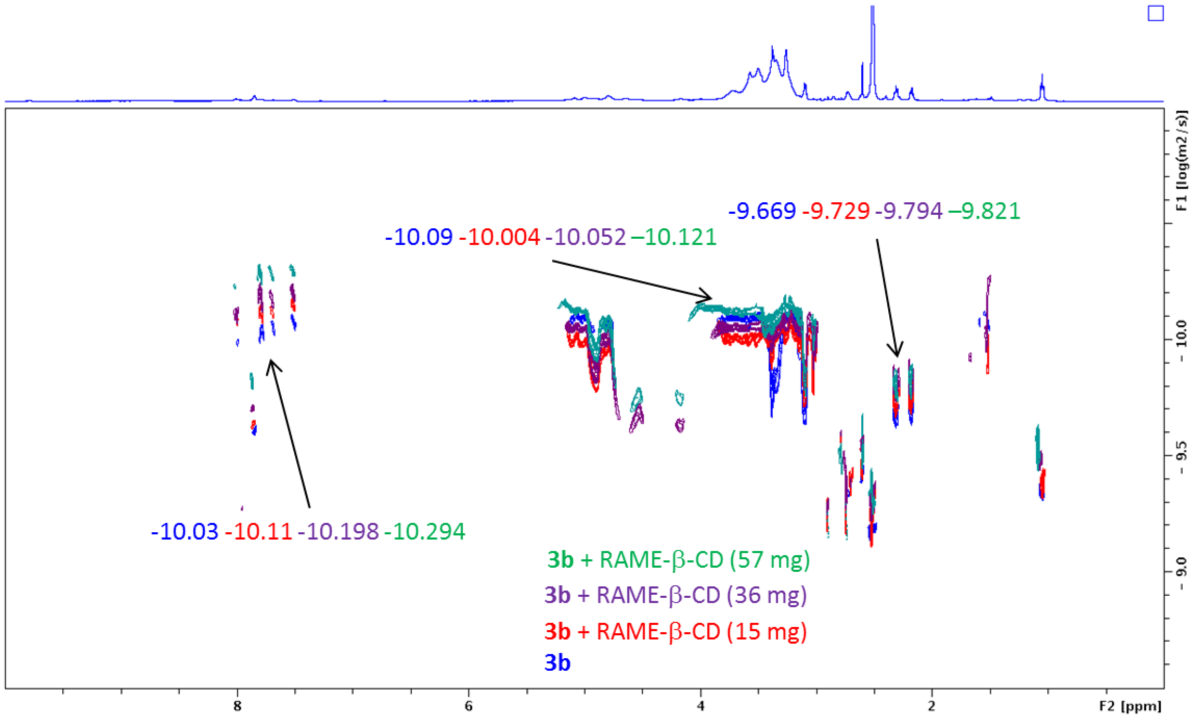

Figure S8. DOSY spectra of **3b** (15 mg) and RAME-β-CD (15 mg) in D_2O (500 µL) at 25 °C.

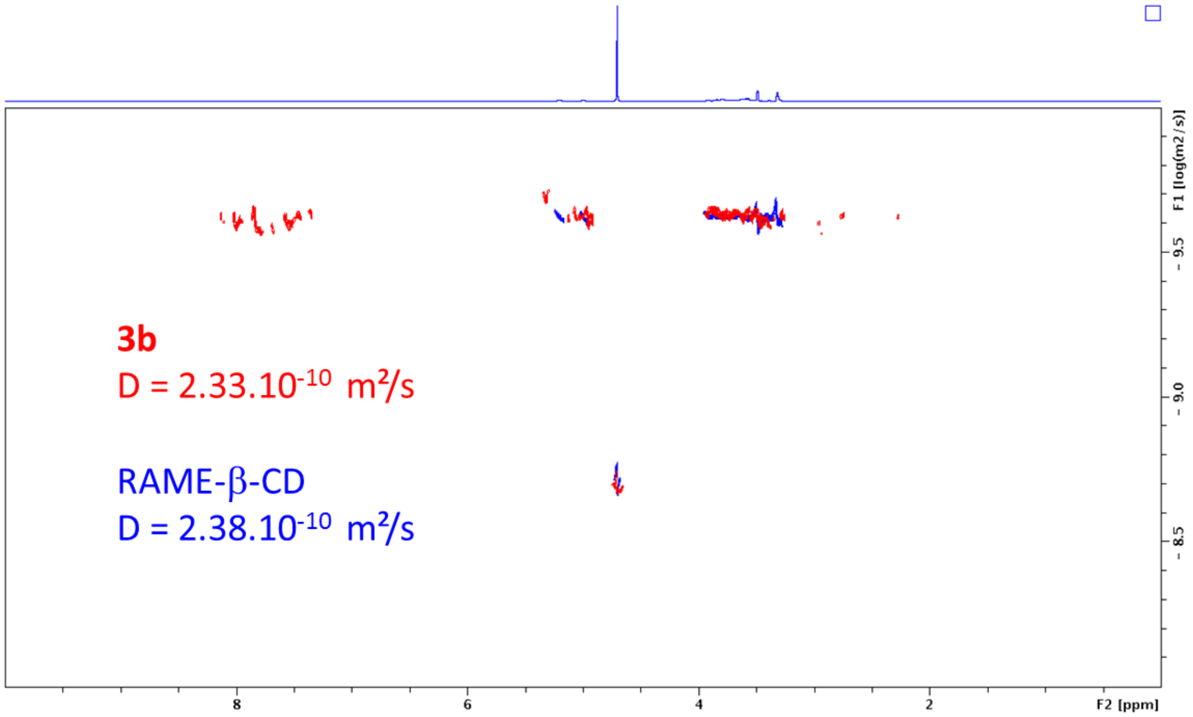

Figure S9. DOSY spectra of mixtures of **3b** (15 mg) and various amounts of RAME-β-CD in D_2O (500 μL) at 25 °C.

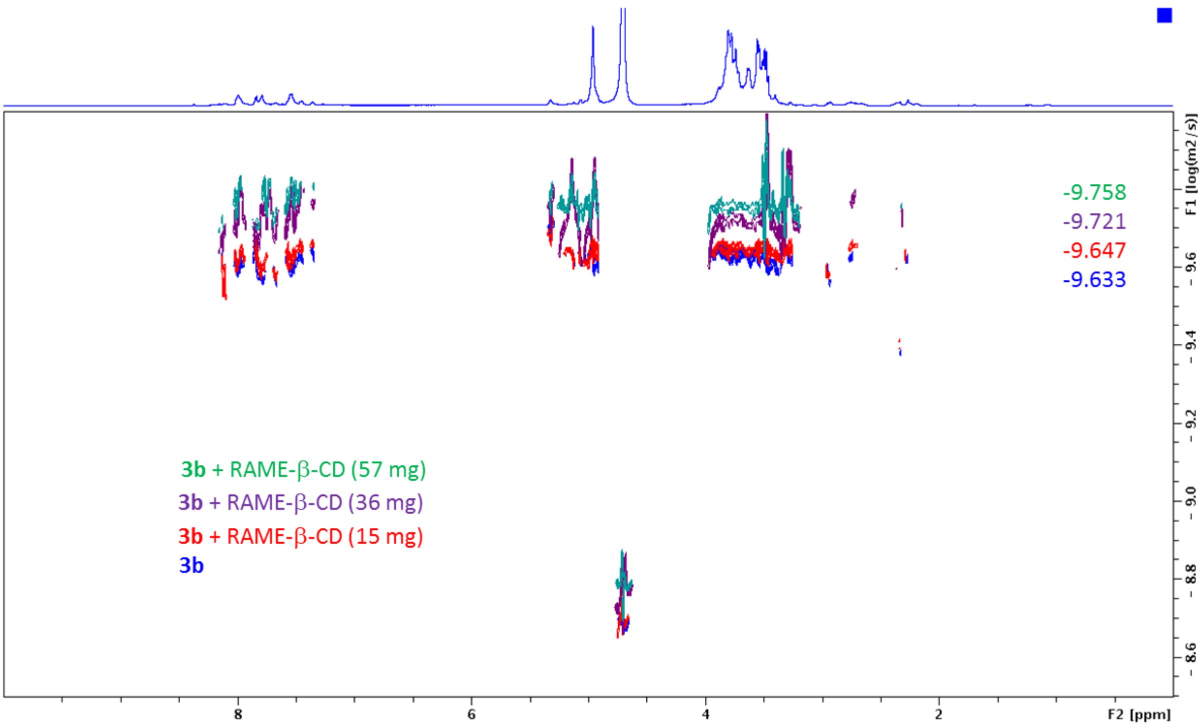

Figure S10. DOSY spectra of **3b** (15 mg) and RAME-β-CD (15 mg) in $CDCl_3$ (500 μL) at 25 °C.

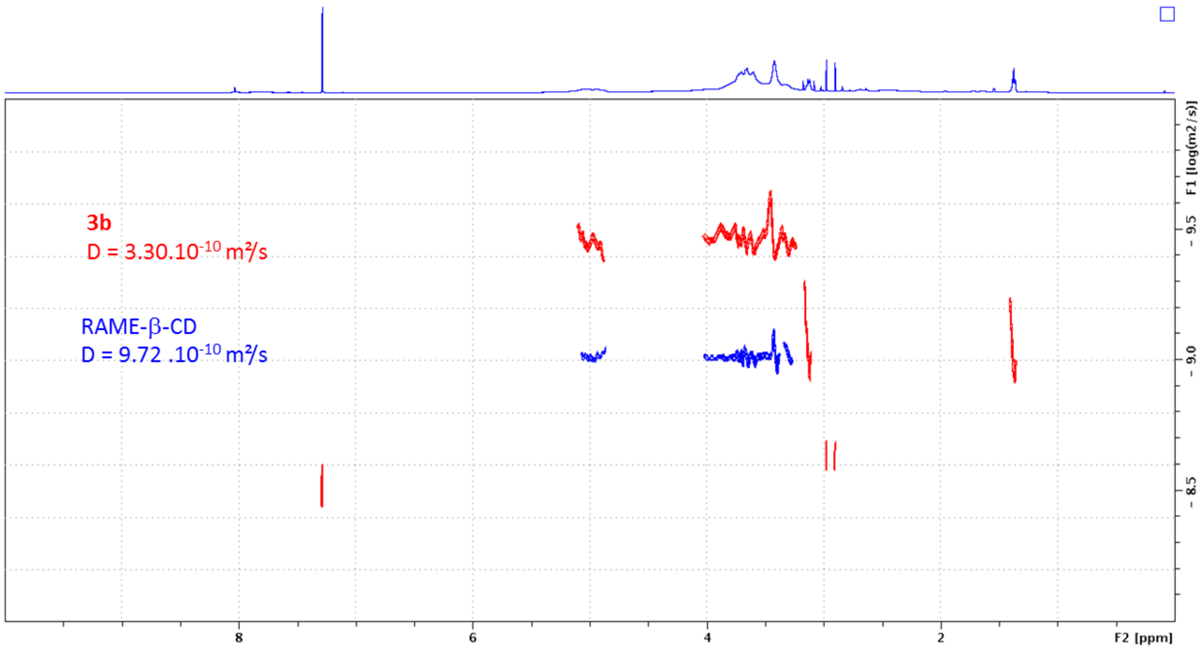

Figure S11. DOSY spectra of **3b** (15 mg) and a mixture of **3b** (15 mg) and RAME-β-CD (15 mg) in $CDCl_3$ (500 μL) at 25 °C.

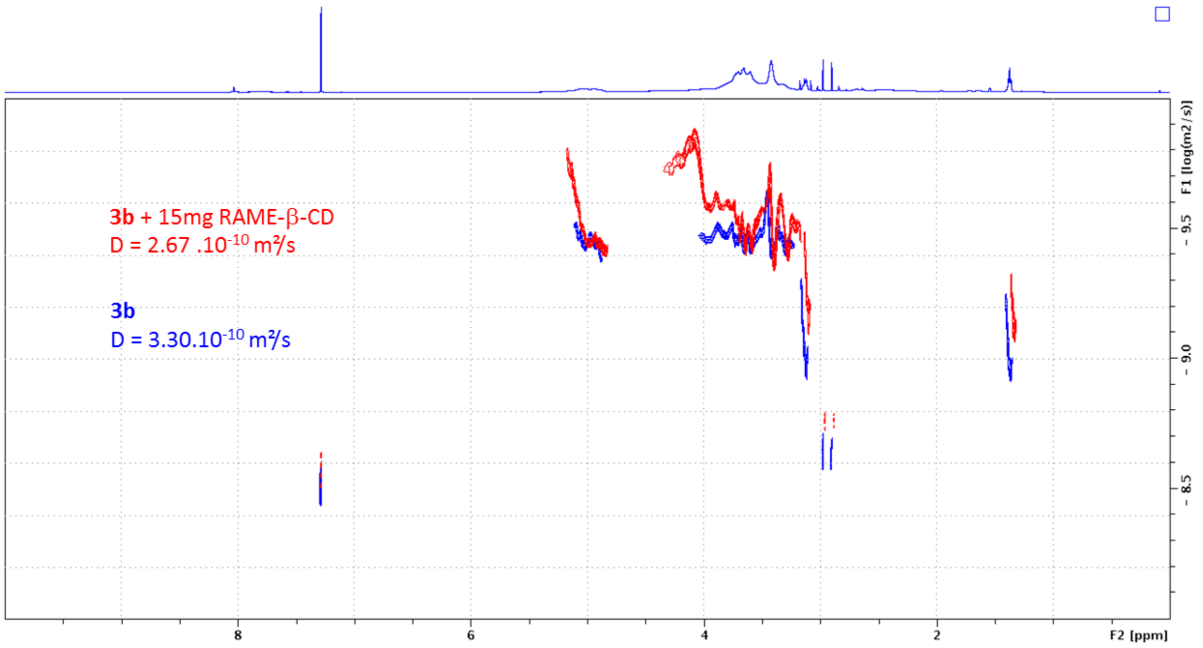

Supplement: File 1 — NMR spectra. [file Beilstein_J_Org_Chem-10-2642-s001.pdf]
